# Enhancing the Bioavailability and Efficacy of Vismodegib for the Control of Skin Cancer: In Vitro and In Vivo Studies

**DOI:** 10.3390/ph15020126

**Published:** 2022-01-21

**Authors:** Heba F. Salem, Amr Gamal, Haitham Saeed, Marwa Kamal, Alaa S. Tulbah

**Affiliations:** 1 Department of Pharmaceutics and Industrial Pharmacy, Faculty of Pharmacy, Beni-Suef University, Beni-Suef 625617, Egypt; Heba_salem111@yahoo.com (H.F.S.); amr_g@pharm.bsu.edu.eg (A.G.); 2 Clinical Pharmacy Department, Faculty of Pharmacy, Beni-Suef University, Beni-Suef 625617, Egypt; haitham.sedawy@pharm.bsu.edu.eg; 3 Clinical Pharmacy Department, Faculty of Pharmacy, Fayoum University, Fayoum 63514, Egypt; mka05@fayoum.edu.eg; 4 Department of Pharmaceutics, College of Pharmacy, Umm Al-Qura University, Makkah 21955, Saudi Arabia

**Keywords:** skin cancer, vismodegib, invasomes, terpenes, bioavailability

## Abstract

Skin cancer is the most frequent cancer throughout the world. Vismodegib (VSD) is a hedgehog blocker approved for the prevention and treatment of skin cancer. VSD, however, is poorly bioavailable and has been linked to side effects. This work focused on designing a nano-invasome gel as a vehicle for enhancing the permeation, bioavailability, and efficacy of VSD. Additionally, the combined effect of terpenes and ethanol was studied on the permeation of VSD compared with liposomes. The prepared VSD-loaded invasomes (VLI) formulation included cineole (1%*v/v*), cholesterol (0.15%*w/w*), phospholipid (2%*w/w*), and ethanol (3%*v/v*) and displayed an entrapment efficiency of 87.73 ± 3.82%, a vesicle size of 188.27 ± 3.25 nm, and a steady-state flux of 9.83 ± 0.11 µg/cm^2^/h. The VLI formulation was vigorously stirred into a carbopol base before being characterized in vivo to investigate the permeation, bioavailability, and efficacy of VSD. The VLI gel enhanced the dermal permeation of VSD and, as a result, had 3.59 times higher bioavailability with excellent antitumor action as compared to oral VSD. In summary, as an alternative to oral administration for skin cancer treatment, invasomes are efficient carriers for delivering VSD and enhancing its transdermal flux into deep skin layers.

## 1. Introduction

Skin cancer is a malignancy that occurs when abnormal cells in the skin proliferate uncontrollably [1,2]. It has now become the most frequent cancer, showing an increasing incidence rate worldwide [3,4]. Vismodegib (VSD) is a hedgehog blocker that has been approved for the prevention and treatment of skin cancer [1,4]. Although oral VSD is effective, it is poorly bioavailable and has been linked to side effects [1,4]. The use of transdermal drug delivery systems has great potential over oral VSD, including avoiding the hepatic first-pass effect, regulating drug delivery, reducing dose frequency, and targeting pathological sites with minimal systemic side effects [5,6]. The human skin’s stratum corneum acts as a protective barrier, limiting the dermal permeation of many drugs. To bypass the stratum corneum, several approaches to transdermal drug delivery systems have been introduced [7]. One of these approaches is to use nanoparticle-based delivery systems.

The use of nanoparticle-based delivery systems has great potential in both clinical and pharmacological research. Drug-loaded nanoparticles allow drugs to penetrate the stratum corneum into the deeper layers of the skin [8]. Nanoparticle-based drug delivery systems improve bioavailability, target action on specific sites, and sustained drug release [9,10]. They can enhance the pharmacokinetics, selectivity, and efficacy of drugs while lowering their toxicity [4,11,12]. Liposomes were first described as nanoparticles for transdermal use [11,13]. Phospholipids and cholesterol are the main components of liposomes. Liposomes can enhance the pharmacokinetics and the efficacy of drugs, but they lack the ability to effectively penetrate the skin [11,13]. A new generation of liposomes was designed to enhance the liposome’s physic-chemical properties. Invasomes were introduced as nanoparticles by Alfred Fahr [14]. They were composed of phospholipids, ethanol, and terpenes [14,15,16]. Ethanol and terpenes are dermal penetration enhancers. Terpenes disturb the stratum corneum’s structure, making the vesicles more fluid [14,15,16]. Ethanol is a –ve charge provider, which makes the lipid membrane more fluidic [5,14,15,16]. The combined effect of ethanol and terpenes enhance drug permeation, bioavailability, and efficacy [14,15,16]. Invasomes are a promising approach to improving transdermal delivery and the permeability of drugs.

Incorporating the invasomes’ suspension into gel as a drug delivery system has great potential for preventing the evaporation of ethanol and terpenes, prolonging the dermal contact time, and improving the drug efficacy, stability, and shelf life [5]. This work focused on designing a nano-invasome gel as a vehicle for enhancing the permeation, bioavailability, and efficacy of VSD. Additionally, the combined effect of terpenes and ethanol was studied on the permeation of VSD compared with liposomes. Formulations of liposomes and invasomes were prepared and characterized in vitro to study their physical and chemical properties. The formulations were then vigorously incorporated into a carbopol gel base as vehicles, before being characterized in vivo to investigate VSD’s permeation, bioavailability, and efficacy.

## 2. Results

### 2.1. HPLC Quantification of Vismodegib

The method developed by Pulusu et al. was used to estimate the calibration curve of VSD. The peak of VSD was evaluated at a retention time of 2.9 min. Linearity was obtained between 0.012 and 0.12 mg/mL with a coefficient of determination (R^2^) of 0.999.

### 2.2. Equilibrium Solubility Studies of Vismodegib

The saturated solubility of VSD was measured and found to be 0.25 mg/mL, and so 50 mL of IPB + Tween 80 (0.1%*v/v*) was great enough to be selected as the dissolution volume to meet the sink condition of the release and permeation experiments.

### 2.3. Preparation and Vitro Characterization of VLI and VLL Formulations

Successfully, the VLI formulation and VLL formulation were prepared as shown in Table 1.

#### 2.3.1. Entrapment Efficiency and DLS Characterization

As shown in Table 1, the %EE, particle size, zeta potential, and polydispersity index (PDI) of the VLL and VLI formulations were measured. The %EE of the VLL formulation was significantly (*p* < 0.05) lower than that of VLI, while the particle size and PDI of the VLI formulation were significantly (*p* < 0.05) lower than those of VLL. The values of the zeta potential of both VLI and VLL formulations indicated enough negative surface charge for electrostatic stabilization.

#### 2.3.2. Thermal Analysis Examination

**Figure 1:**
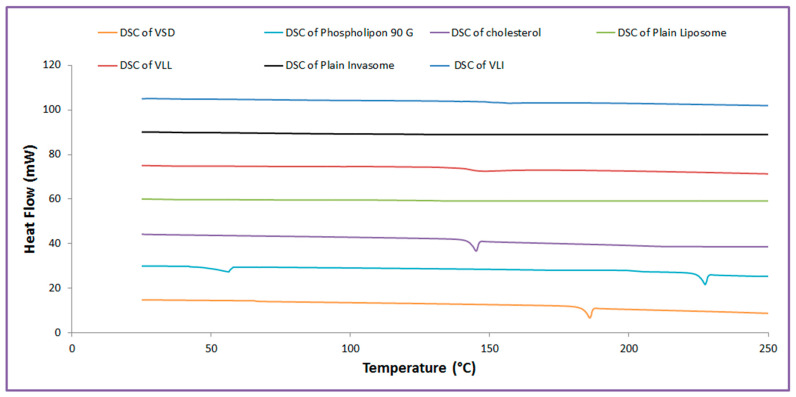
Thermal analysis of VLI and VLL formulations.

As shown in Figure 1, VSD, phospholipid, and cholesterol showed endothermic peaks at 186 °C, 224 °C, and 144.46 °C, respectively. The DSC thermogram of plain liposome, plain invasomes, VLI, and VLL shows the disappearance of the endothermic peaks.

#### 2.3.3. Transmission Electron Microscopy (TEM) Measurement

**Figure 2:**
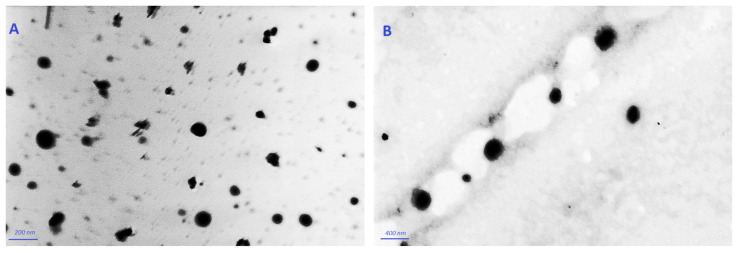
TEM micrographs of VLI (**A**) and of VLL (**B**).

According to the TEM, Figure 2 displays the VLI and VLL formulations’ surface morphology. Both formulations appear as spherical nano-vesicles with black dots.

### 2.4. Preparation and In Vitro Characterization of Gel Formulations

#### 2.4.1. Preparation and Characterization of VLI and VLL Gel Formulations

As shown in Table 2, the carbopol gel base was successfully blended with the VLI and VLL formulations. The results showed that the prepared VLL, VLI, and free VSD gel formulations showed viscosity coefficients of 154.08 ± 1.54, 136.34 ± 1.92 cP, and 166.82 ± 2.14 cP, respectively.

#### 2.4.2. Ex Vivo Permeability and Disposition Studies

**Figure 3:**
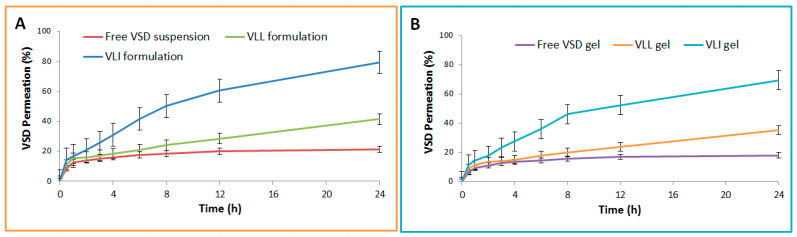
(**A**) Ex vivo permeation profiles of free VSD suspension and VLI and VLL formulations. (**B**) Ex vivo permeation profiles of free VSD and VLI and VLL gel formulations.

As shown in Table 2, the steady-state (Fss) and skin disposition of free VSD suspension, VLI formulation, VLL formulation, free VSD gel, VLL gel, and VLI gel were measured. The permeation and Fss of the VLL formulation were significantly (*p* < 0.05) lower than those of the VLI formulation, while the skin disposition of the VLI formulation was significantly (*p* < 0.05) lower than that of the VLL formulation. Figure 3 shows that the permeation of VLI and VLL formulations was significantly (*p* < 0.05) retarded upon their incorporation into the gel formulation.

#### 2.4.3. In Vitro Drug Release Kinetic Studies

**Figure 4:**
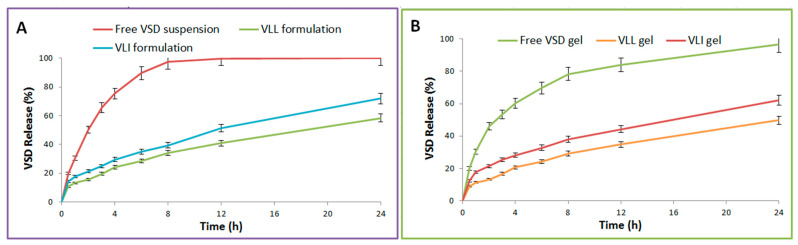
(**A**) In vitro release profiles of free VSD suspension and VLI and VLL formulations. (**B**) In vitro release profiles of free VSD and VLI and VLL gel formulations.

As shown in Table 2, the release of free VSD suspension, VLI formulation, VLL formulation, free VSD gel, VLL gel, and VLI gel was measured. The release of the VLL formulation was significantly (*p* < 0.05) lower than that of the VLI formulation. Figure 4 shows that the release of VLI and VLL formulations was significantly (*p* < 0.05) retarded upon their incorporation into the gel formulation.

DDSolver software was used to evaluate the in vitro release kinetics and mechanism of VSD from the VLI gel formulation. The Higuchi model was selected to fit the release data due to its maximum R^2^ (0.9974), MSC (5.2340), and minimum AIC (23.3039). As the calculated “n” was 0.421 ± 0.02, the Fickian diffusion was the release mechanism of VSD from the VLI gel formulation. Additionally, f_2_ was calculated and found to be 24.38 ± 1.73, indicating a significant (*p* < 0.05) difference in dissolution profiles.

### 2.5. In Vivo Anti-Tumor Characterization of VLI Gel Formulation

#### 2.5.1. Anti-Tumor Activity of VLI Gel Formulation

**Figure 5:**
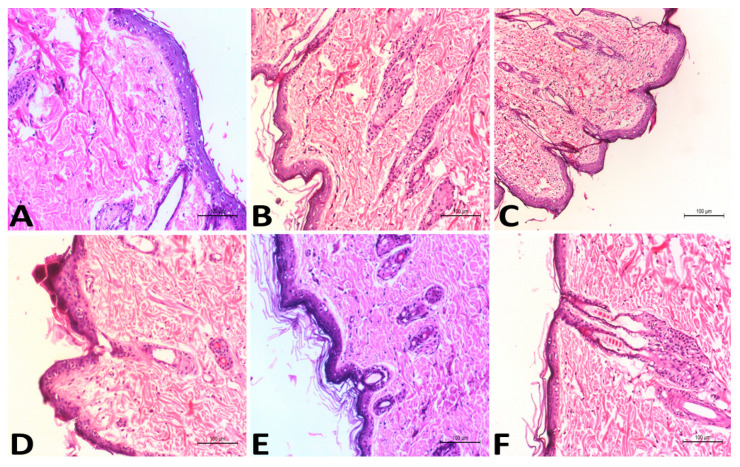
Histopathological studies of the negative control group (**A**), positive control group (**B**), groups treated with free VSD gel (**C**), oral VSD (**D**), VLL gel (**E**), and VLI gel (**F**).

Compared to the group treated with oral VSD, the number and diameter of papillomas of the group treated with the VLI gel were significantly (*p* < 0.05) decreased. To confirm these results, the histopathology of each treatment was studied. The presence of neoplastic proliferative epithelial cells was seen histopathologically in the positive control group (Figure 5B) with dermal edema, hyperkeratosis, and inflammatory cell infiltrations. In the group treated with the free VSD gel, there was no improvement in the symptoms of skin toxicity in the dermis or epidermal layers (Figure 5C). The presence of hyperkeratosis in the epidermis was seen histopathologically in the group treated with oral VSD (Figure 5D) with dermal edema and diffused inflammatory reaction. A marked reduction in the hyperplasia and keratosis was seen histopathologically in the group treated with the VLL gel (Figure 5E), with a marked improvement in the size and number of papillomas. The group treated with the VLI gel (Figure 5F) showed excellent effects on the different skin layers, with the absence of all signs of skin toxicity. Skin papillomas were revealed, and hyperplasia and hyperkeratosis were absent.

#### 2.5.2. Toxicity of VLI Gel

**Figure 6:**
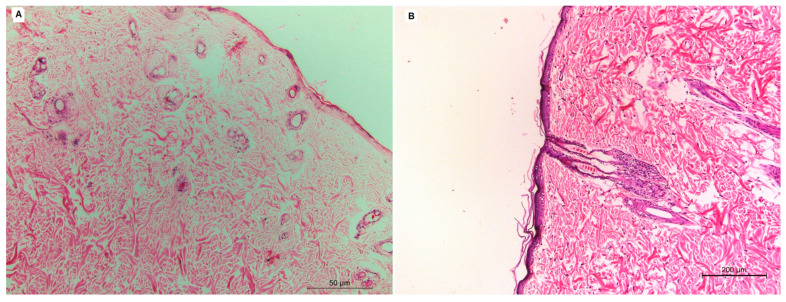
Histopathological studies of the negative control group (**A**) and group treated with VLI gel (**B**).

The histopathology of the group treated with the VLI gel (Figure 6B) was studied and compared with that of the negative control group (Figure 6A). There was not any inflammatory cell infiltration observed in the dermal layers of the group treated with the VLI gel, showing nearly normal skin similar to that of the negative control group.

#### 2.5.3. In Vivo Permeation and Bioavailability Studies

**Figure 7:**
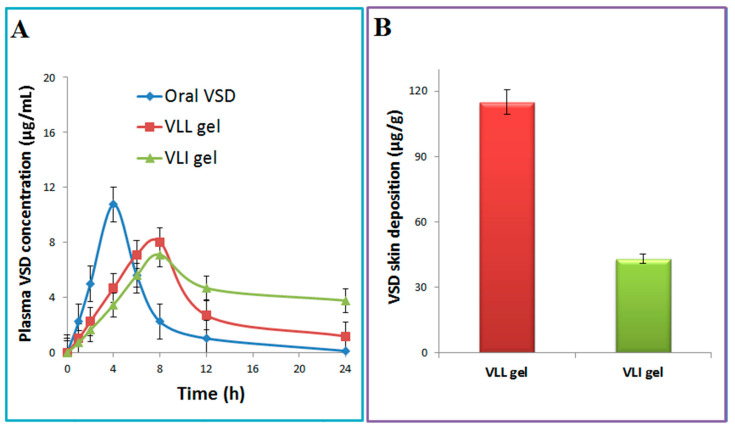
VSD concentration in plasma (**A**) and skin (**B**) after administration of oral VSD, VLI gel, and VLL gel.

PK-SOLVER was used to draw the relation between plasma concentration and time (Figure 7A). As shown in Table 3, the AUC for the VLI gel was significantly (*p* < 0.05) greater than the AUC of the VLL gel by 1.56 fold and the AUC of oral VSD by 3.59 fold. The relative bioavailability of the VLI gel formulation relative to oral VSD and VLL gel formulation was 3.59 and 1.56, respectively. Compared to the oral VSD suspension, the VLL gel showed lower Cmax and longer Tmax. In addition, the t_0.5_ and MRT for the VLL gel were significantly (*p* < 0.05) higher than those of the oral VSD suspension.

#### 2.5.4. In Vivo Skin Disposition Studies

Compared with the VLL gel, the concentration of VSD deposited in the skin after VLI gel was measured to confirm the enhancement effect of terpenes and ethanol on the liposome’s permeation. Figure 7B shows a significantly (*p* < 0.05) higher skin concentration for the VLL gel compared with that of the VLI gel.

## 3. Discussion

New transdermal techniques have been explored to improve the drug’s permeation and bioavailability [17]. Invasomes are a promising approach to improving the transdermal delivery, permeability, and efficacy of drugs [14,18,19,20,21]. In this study, the combined effect of ethanol and terpenes was investigated to enhance the permeation of VSD, bioavailability, and efficacy. Invasomes are made of phospholipids, cholesterol, ethanol, and terpenes. Preliminary trials were conducted to identify the concentration ranges of independent variables. Phospholipids are the building blocks of lipid bilayers [5,21]. Preliminary trials revealed that phospholipid has a synergistic effect on the %EE and particle size up until a certain concentration was reached, at which point further phospholipid increment produced larger vesicles with no effect on the %EE [14]. Cholesterol provides rigidity and stability to the lipid bilayer [22,23]. In addition, preliminary trials revealed that cholesterol has a synergistic effect on the %EE and stability until a certain concentration is reached, at which point cholesterol competes with the drug and decreases the %EE [5]. Ethanol is a penetration enhancer and negative charge provider, but increasing the ethanol concentration produced a leaky and more fluidic lipid membrane, conveying the entrapped drug out of the vesicles [7]. According to literature reviews, invasomes including cineole (1%*v/v*) are efficient carriers for delivering drugs and enhancing their transdermal flux into deep skin layers [14,19]. Successfully, a VLI formulation including cineole (1%*v/v*), cholesterol (0.15%*w/w*), phospholipid (2%*w/w*), and ethanol (3%*v/v*) was prepared.

The amount of entrapped VSD in the vesicular systems was calculated, and the %EE of the VLL formulation was significantly (*p* < 0.05) lower than that of the VLI formulation. The presence of ethanol and terpene in the invasomal lipid bilayer disrupted the hydrogen bonds between the ceramides in the skin, increasing the space available for drug incorporation [15,24]. As vesicles must penetrate the skin, vesicle size is a crucial factor to consider when evaluating the formulation [15,16]. The particle size of the VLI formulation was significantly (*p* < 0.05) lower than that of the VLL formulation due to the steric repulsion among terpene molecules [25]. Additionally, ethanol reduces vesicle aggregation by increasing the negative charges of vesicles and electrostatic repulsion [1,11]. The polydispersity index analysis of the prepared formulations revealed a low polydispersity index, which supported the presence of homogeneity and a narrow distribution of particle size. The prepared formulations possessed a negative surface charge, indicating good physical and chemical stability, enough for electrostatic stabilization [20]. The DSC analysis was performed to determine the melting point, compatibility of vesicles’ components, and how much a material’s enthalpy had changed over time as a result of changes in its physical and chemical properties [11,26]. The crystallinity of cholesterol and phospholipon 90 G was diminished upon mixing together due to the lipid bilayer formation. The crystallinity of VSD was diminished upon mixing because VSD is a highly hydrophobic drug, which appeared in an amorphous form.

Carbopol^®^ is an anionic polymer that has an extremely high buffering capacity and does not irritate the skin [27,28]. The carbopol^®^ polymer has the required viscosity and bio-adhesive properties [5,28]. Compared with the free VSD gel formulation, ethanol and cineole were responsible for the VLI gel formulations’ lower viscosity coefficients. The dissolution volume was selected to suit the VSD’s sink condition. The permeation of VSD from the VLI formulation was significantly higher due to the synergistic effect of ethanol and terpenes [29,30]. When invasomes are applied to the skin, one part of the vesicle disintegrates and releases its components, such as terpenes, phospholipid, and ethanol, which enhance the penetration and fluidize the stratum corneum [15,31]. Furthermore, the small size of invasomes increases the surface area, the interaction with the stratum corneum, and, consequently, the drug permeation [29]. Due to the cross-linking of carbopol gel, incorporating VLI into it considerably slowed the release and permeation of VSD [5,28]. DDSolver software revealed a significant difference in dissolution profiles, indicating that invasomes delayed VSD release [16].

VLI gel had improved bioavailability and anti-tumor activity when compared to oral VSD and VLL gel at the same dose due to the enhancement effect of terpenes on the VSD’s permeation. Additionally, histopathological examination revealed that the VLI gel had no toxicity at the dose regimen used. The bioavailability of the VLI gel was greater than that of oral VSD due to avoiding hepatic first-pass metabolism and the enhancement effect of terpenes on the VSD’s permeation. When compared to oral VSD, the VLI gel’s Tmax and Cmax revealed a regulated drug release and lowered the undesired VSD concentration. Furthermore, the longer blood circulation time was reflected by a significant increase in MRT and t_0.5_ for the VLI gel. Invasomes improved the specificity of VSD in delivery and targeting, lowering the toxicity of VSD because invasomes act as a reservoir of the drug at the site of application, providing a controlled and sustained delivery of the drug. When compared to the VLL gel, the VLI gel’s AUC, MRT, and t_0.5_ revealed the enhancement effect of terpenes on the VSD’s permeation. Due to the synergistic action of both ethanol and terpenes, the VLI gel had a much lower skin disposition concentration. As alcoholic –OH is more electronegative than amide NH, terpenes break hydrogen bridges that bind ceramides in the skin [24,31,32].

Previous preclinical and clinical studies employed different oral doses of VSD, ranging from 1 to 10 mg/kg [1,4,33,34]. These studies showed that VSD has low bioavailability, which was also reported by the current study using 10 mg/kg. Application of the VLI gel formulation resulted in enhanced bioavailability by 3.59 fold and, consequently, resulted in significant anti-tumor activity compared to oral VSD. Previously, various topical formulations of VSD have been developed to improve skin localization and efficacy in comparison to oral vismodegib [1,4,35]. Calienni et al. developed vismodegib-ultradeformable liposomes as a topical preparation. The results showed that the cytotoxicity and performance of vismodegib were enhanced [35]. Other researchers found that the Fss of vismodegib from ethosomal gel was higher than that of binary ethosomes and positively charged ethosomes [1,4]. Our work focused on designing a VSD-loaded nano-invasome system to study the effect of terpenes on enhancing VSD’s permeation, bioavailability, and efficacy. The higher Fss of VLI gel is attributed to the higher relative bioavailability and excellent effects of the vismodegib-loaded invasomes gel formulation.

## 4. Materials and Methods

### 4.1. Materials

Vismodegib standard was attained by the National Research Center (Egypt) as a gift. Cineole, ethanol, phospholipid (phospholipon 90 G), and 7,12-dimethylbenz(a)anthracene (a tumor initiator, DMBA) were supplied by Agitech Pharmaceutical Company (Cairo, Egypt). Methanol, carbopol^®^, and acetonitrile were supplied by Cornell Lab in Egypt.

### 4.2. HPLC Quantification of Vismodegib

VSD quantification was carried out using the HPLC method (Waters 2690 Alliance HPLC system), as described by Pulusu et al. [36]. The assay of VSD was carried out using a C-18 column ZORBAX with 150 × 4.6 mm dimensions. A solution of acetonitrile and 0.1% orthophosphoric acid at a ratio of 50:50 *v/v* was prepared as a mobile phase. The detection of VSD was processed in triplicates at a maximum wavelength of 264 nm, with a flow rate of 1 mL/min and an injection volume of 10 µL.

### 4.3. Equilibrium Solubility Study

The equilibrium solubility study was conducted to estimate the VSD’s saturation solubility and the dissolution volume required to meet the sink conditions of the prepared formulations’ permeation and release studies [4]. VSD was vigorously stirred for 3 days at 37 °C. The solution was filtered and the VSD content was calculated using HPLC in triplicates.

### 4.4. Preparation of Vismodegib-Loaded Invasomes and Vismodegib-Loaded Liposomes

A vismodegib-loaded invasomes (VLI) formulation was prepared, as described by Shah et al., by a method of thin hydration [18]. A chloroformic solution of VSD (10 mg), cineole (1%*v/v*), cholesterol (0.15%*w/w*), and phospholipid (2%*w/w*) was obtained and evaporated under vacuum conditions (100 rpm, 40 °C). A solution of isotonic phosphate buffer (IPB, pH 5.5) and ethanol (3%*v/v*) was obtained to hydrate the produced lipid film at 40 °C for 1 h. The prepared VLI formulation was sonicated and stored at 4 °C.

The thin hydration method was used to prepare a vismodegib-loaded liposomes (VLL) formulation [11]. A chloroformic solution of VSD (10 mg), cholesterol (0.15%*w/w*), and phospholipid (2%*w/w*) was obtained and evaporated under vacuum conditions (100 rpm, 40 °C). A solution of isotonic phosphate buffer (IPB, pH 5.5) was obtained to hydrate the produced lipid film at 40 °C for 1 h. The prepared VLL formulation was sonicated and stored at 4 °C.

### 4.5. In Vitro Evaluation of VLI and VLL Formulations

#### 4.5.1. Entrapment Efficiency Measurement

VSD content was determined by centrifuging (SIGMA, Steinheim, Germany) a sample of the prepared VLI and VLE formulations at 20,000 rpm for 1 h [37]. The VLI and VLE pellets were collected and lysed in methanol. The collected supernatant and methanolic solution were diluted with the mobile phase and the %EE was calculated using HPLC in triplicates as follows [26]:

%EE = [(At − S)/At] × 100 (1)

where At is the total amount of VSD in the supernatant and pellets, S is the amount of VSD in the supernatant. The calculated %EE of the VLL and VLI formulations was subjected to a Student’s *t*-test at *p* < 0.05.

#### 4.5.2. Vesicle Size and Zeta Potential Measurement

Dynamic Light Scattering (DLS) of a sample of the VLI and VLL formulations was obtained using a Zetasizer (Malvern, Herrenberg, Germany) to characterize their size and polydispersity index (PDI) in triplicates [38]. Zeta potential was also characterized using a Malvern Zetasizer. The estimated parameters of the Zetasizer of the VLL and VLI formulations were subjected to a Student’s *t*-test at *p* < 0.05.

#### 4.5.3. Thermal Analysis Examination

Differential Scanning Calorimetry (DSC) of a sample of cholesterol and phospholipid, VSD, plain invasomes, plain lipomes, and VLI and VLL formulations was obtained using a calorimeter (NETZSCH-Geratebau GmbH, Maia, Germany) to characterize their thermal analysis [1]. With a nitrogen flow rate of 25 mL/min, DSC thermograms were carried out at a rate of 5 °C/min. The samples were promptly cooled to 25 °C after being heated to 250 °C.

#### 4.5.4. Transmission Electron Microscopy (TEM) Measurement

A sample of the VLI and VLL formulations was deposited onto a carbon-coated copper grid and visualized by Transmission Electron Microscopy (TEM, Carl Zeiss, Oberkochen, Germany) at suitable magnifications [13].

### 4.6. Preparation and In Vitro Characterization of Gel Formulations

#### 4.6.1. Incorporation of VLI and VLL Formulations into Carbopol Gel Base

VLI, VLL, and free VSD gel formulations were created by incorporating the VLI, VLL, and free VSD into 2%*w/w* carbopol 974 gel base [5]. Carbopol 974 was vigorously stirred in water, and triethanolamine was added to alter the pH of the gel base. VLI, VLL, and free VSD were vigorously stirred into the carbopol gel base.

#### 4.6.2. Viscosity Coefficient Determination

The Brookfield viscometer (Brookfield DV-III ULTRA, Middleborough, MA, USA) was used to measure the viscosity coefficient in triplicates of VLI, VLL, and free VSD gel formulations [4]. The calculated viscosity coefficient of VLI, VLL, and free VSD gel formulations was subjected to one-way ANOVA test with a Bonferroni post hoc test at *p* < 0.05.

#### 4.6.3. Ex Vivo Permeability and Skin Disposition Studies

The studied samples were free VSD suspension, VLI formulation, VLL formulation, free VSD gel formulation, VLL gel formulation, and VLI gel formulation. Samples (equivalent to 1 mg VSD) were loaded onto guinea pig skin (5 cm^2^) as a diffusion membrane [4]. At 100 rpm and 37 ± 0.5 °C, 50 mL of IPB and Tween 80 (0.1%*v/v*) was used as the dissolution medium. At predefined time points, samples of 3 mL were taken and replaced with an equal volume. Samples were diluted with a mobile phase and the VSD content was calculated using HPLC to measure the cumulative amount permeated (µg/cm^2^) and the steady-state flux (Fss) (µg/cm^2^/h) in triplicates. The estimated parameters of permeability studies were subjected to an ANOVA test with a Bonferroni post hoc test at *p* < 0.05.

At the end of the experiment, guinea pig skin from the studied samples was collected and dipped in water at 60 °C for 45 s. The skin was homogenized at 8000 rpm for 10 min and the resulting product was filtered [39]. The amount of VSD disposed of in the skin from the studied samples was calculated using HPLC in triplicates. The calculated VSD disposed of was subjected to an ANOVA test with a Bonferroni post hoc test at *p* < 0.05.

#### 4.6.4. In Vitro Release Studies

The studied samples were free VSD suspension, VLI formulation, VLL formulation, free VSD gel formulation, VLL gel formulation, and VLI gel formulation. Samples (equivalent to 1 mg VSD) were loaded on a dialysis bag as a diffusion membrane [40]. At 100 rpm and 37 ± 0.5 °C, 50 mL of IPB and Tween 80 (0.1%*v/v*) was used as the dissolution medium. At predefined time points, samples of 3 mL were taken and replaced with an equal volume. Samples were diluted with a mobile phase and the VSD content was calculated using HPLC to measure the %VSD released in triplicate. The %VSD released was subjected to an ANOVA test with a Bonferroni post hoc test at *p* < 0.05.

#### 4.6.5. Kinetic Analysis of Release Data

DDSolver program software was used to determine the kinetics of VSD’s release from the VLI gel formulation [41,42]. The DDSolver selects the model that fits the VSD’s release data according to the criteria of achieving the minimum Akaike information criterion (AIC) and the highest coefficient of determination (R^2^) and model selection criterion (MSC) criteria. In addition, DDSolver determines the mechanism of VLI’s release using the Korsmeyer–Peppas equation [43]. The mechanism is Fickian diffusion if *n* = 0.5 and is non-Fickian diffusion if 0.5 < *n* < 1. Furthermore, the DDSolver calculates the similarity factor “f_2_” to determine the significance (*p* < 0.05) of the difference in dissolution profiles between the VLI formulation and free VSD suspension using the Student’s *t*-test at *p* < 0.05. The difference is insignificant if f_2_ > 50 and is significant if f_2_ < 50.

### 4.7. In Vivo Anti-Tumor Characterization of VLI Gel Formulation

#### 4.7.1. Animal Study

Fifty adult male rats (200–300 g) were placed in accordance with the guidelines of ethical approval of the Faculty of Pharmacy, Beni-Suef University, Beni-Suef, Egypt (BSU-IACUC 021). Before beginning the experiment, the skin of each rat was trimmed within a 3 cm × 3 cm area. To initiate the tumor, each rat was subcutaneously injected with 1 mg of DMBA [44]. Malignant tumors known as papillomas were observed. Rats were divided into 6 groups at random and the treatment was administered as follows:

Group 1  Negative control group
Group 2  Positive control group
Group 3  Free VSD suspension
Group 4  Free VSD gel
Group 5  VLL gel formulation
Group 6  VLI gel formulation

The gel formulations were topically set on the backs of the rats three times per week at a dose of 10 mg/kg body weight. A stomach tube gavage was used to administer the oral free VSD suspension.

#### 4.7.2. Anti-Tumor Activity and Toxicity of VLI Gel Formulation

The papilloma number and diameter of each rat were recorded weekly to measure the efficacy of each treatment [44]. At the end of the experiment, each rat was anesthetized by inhaled sevoflurane and sacrificed. The tumor samples were taken and fixed in formalin for histopathological examination. A histopathology investigation was used to examine the anti-tumor activity and toxicity of each treatment [45].

#### 4.7.3. In Vivo Permeation and Bioavailability Studies

These studies were obtained to investigate the combined effect of terpenes and ethanol on the permeation and bioavailability of VSD compared with those of oral VSD suspension and VLL gel. Eighteen adult male rats weighing 200–300 g were divided into three groups (*n* = 6). The first group was provided a free VSD suspension to take orally. The VLL gel formulation was topically applied to the second group. The VLI gel formulation was topically applied to the third group. At predefined time points over 24 h, blood samples (200 µL) were taken from the retro-orbital sinus and centrifuged [7]. The supernatant was vaporized and dissolved in the mobile phase before being analyzed by HPLC [46]. The non-compartmental analysis using PK-SOLVER was used to calculate the pharmacokinetic parameters such as C_max_ and T_max_ from the graph curve. The trapezoid rule was used to determine the areas under the curves (AUC_0-t_ and AUC_0-inf_). The slope of the log plasma concentration vs. time plot was used to calculate the elimination rate constant (K). Using the formula t_0.5_ = 0.693/K, the elimination half-life (t_0.5_) was computed. The estimated parameters of three groups were subjected to the one-way ANOVA test with a Bonferroni post hoc test at *p* < 0.05.

#### 4.7.4. In Vivo Skin Disposition Studies

At the end of the in vivo permeation experiment after 24 h, skin sections from the second and third groups were taken and the stratum corneum was removed by the tape stripping technique [47]. The skin was collected and dipped in water at 60 °C for 45 s. The skin was homogenized at 8000 rpm for 10 min and the resulting product was filtered. The supernatant was vaporized and dissolved in the mobile phase before being analyzed by HPLC. The VSD content was calculated to measure the skin deposition of the VLI gel and the VLL gel in triplicates. The estimated parameters were subjected to a Student’s *t*-test at *p* < 0.05.

## 5. Conclusions

The vismodegib-loaded invasomes formulation that included cineole (1%*v/v*), cholesterol (0.15%*w/w*), phospholipid (2%*w/w*), and ethanol (3%*v/v*) was prepared and displayed an entrapment efficiency of 87.73 ± 3.82%, a vesicle size of 188.27 ± 3.25 nm, and a steady-state flux of 9.83 ± 0.11 µg/cm^2^/h. The VLI formulation was vigorously stirred into the carbopol base before being characterized in vivo to investigate the permeation, bioavailability, and efficacy of VSD. The VLI gel enhanced the dermal permeation of VSD and, as a result, had 3.59 times higher bioavailability alongside excellent antitumor action as compared to oral VSD. In summary, as an alternative to oral administration for skin cancer treatment, invasomes are efficient carriers for delivering VSD and enhancing its transdermal flux into deep skin layers.

## Figures and Tables

**Table 1:** In vitro characterization of VLI and VLL formulations.

Formulation Code	Phospholipid %*w/w*	Cholesterol %*w/w*	Cineole %*v/v*	Ethanol %*v/v*	%EE (%) (Mean ± SD)	Particle Size (nm) (Mean ± SD)	Zeta Potential (mV) (Mean ± SD)	PDI (Mean ± SD)
VLI	2	0.15	1	3	87.73 ± 3.82	188.27 ± 3.25	−20.5 ± 1.61	0.12 ± 0.02
VLL	2	0.15	0	0	72.52 ± 4.72	258.67 ± 9.00	−7.72 ± 1.53	0.26 ± 0.02

**Table 2:** In vitro release and ex vivo permeation studies of free VSD, VLI, and VLL gel formulations.

Formulation Code	Release (%) (Mean ± SD)	Steady-State Flux (µg/cm^2^/h) (Mean ± SD)	Skin Disposition (µg/cm^2^) (Mean ± SD)
Free VSD suspension	99.60 ± 0.96	1.35 ± 0.03	147.23 ± 3.18
VLL	58.55 ± 1.04	2.73 ± 0.09	106.86 ± 2.98
VLI	71.96 ± 0.86	9.83 ± 0.11	31.09 ± 2.68
Free VSD gel	96.60 ± 1.24	1.17 ± 0.07	153.97 ± 3.95
VLL gel	49.76 ± 0.88	2.43 ± 0.06	119.56 ± 4.40
VLI gel	62.12 ± 1.27	9.14 ± 0.09	51.27 ± 4.84

**Table 3:** Pharmacokinetic parameters after administration of oral VSD, VLI gel, and VLL gel. * VLI gel was significant with VLL gel at *p* < 0.05 by one-way ANOVA–Bonferroni post hoc test; # VLI gel was significant with oral VSD at *p* < 0.05 by one-way ANOVA–Bonferroni post hoc test.

Pharmacokinetic Parameters	VLI Gel	VLL Gel	Oral Free VSD Suspension
Cp_max_ (μg/mL)	7.11 ± 0.82 #	8.02 ± 0.66	10.76 ± 0.57
T_max_ (h)	8	8	4
AUC_(0–24)_ (μg.h/mL)	102.70 ± 5.69 *,#	80.82 ± 5.03	58.18 ± 4.03
AUC_(0–inf)_ (μg.h/mL)	211.34 ± 9.70 *,#	91.83 ± 7.13	58.86 ± 3.83
AUMC_(0–inf)_ (μg.h/mL)	7002.80 ± 135.41 *,#	1119.99 ± 112.32	361.34 ± 30.19
MRT (h)	33.13 ± 2.13 *,#	12.19 ± 2.33	6.13 ± 2.17
t_0.5_ (h)	19.98 ± 1.85 *,#	6.43 ± 1.05	3.83 ± 0.85

## Data Availability

Data is contained within the article.
